# Supplementary material for: Electronically steered metasurface antenna
Source: Sci Rep. 2021 Feb 25;11:4693. doi: 10.1038/s41598-021-83377-9 (PMC7907413; doi:10.1038/s41598-021-83377-9)
Supplement: Supplementary file 1 — Supplementary Information 1. [file 41598_2021_83377_MOESM1_ESM.pdf]

Electronically Steered Metasurface Antenna

Michael Boyarsky*, Timothy Sleasman, Mohammadreza F. Imani, Jonah N. Gollub, and David R. Smith

Center for Metamaterials and Integrated Plasmonics
Department of Electrical and Computer Engineering
Duke University, Durham, NC 27708, USA

*Corresponding author: michaelboyarsky@gmail.com

Supplementary Materials

Included with the above manuscript are the following two video files:

File 1: ‘supplemental_circular_steering.avi’

This file contains a video showing the metasurface antenna electronically steering around two circular paths. The first circular path starts at −15° and the second circular path starts at −25°.

File 2: ‘supplemental_linear_steering.avi’

This file contains a video showing the metasurface antenna electronically steering in azimuth then in elevation. These beam patterns correspond with the cross section radiation patterns shown in Figures 7 and 8 in the manuscript.
